# Activities and repercussions of a course on Sustainable Development Goals with a focus on surveillance

**DOI:** 10.1590/0034-7167-2024-0405

**Published:** 2025-10-27

**Authors:** Camila Rodrigues Barbosa Nemer, Lethicia Barreto Brandão, Matheus Lopes dos Santos, Gabriella Ferreira Gomes, Anneli Mercedes Celis de Cárdenas, Marta Azevedo dos Santos

**Affiliations:** I Universidade Federal do Amapá. Macapá, Amapá, Brazil; II Universidade Federal do Tocantins. Palmas, Tocantins, Brazil

**Keywords:** Sustainable Development, Noncommunicable Diseases, Public Health Surveillance, Primary Health Care, Health Promotion, Desarrollo Sostenible, Enfermedades no Transmisibles, Vigilancia en Salud Pública, Atención Primaria de Salud, Promoción de la Salud

## Abstract

**Objectives:**

to describe the activities and repercussions of training course “Sustainable Development Goals with a focus on Surveillance of Noncommunicable Diseases and Injuries in the Legal Amazon”.

**Methods:**

an observational and descriptive study. Thirty-six healthcare professionals from 14 municipalities in the state of Amapá, AP, Brazil participated. Preand post-tests and observations (non-participant) were applied.

**Results:**

during the course, welcoming activities and educational and theoretical practices were developed to improve participants’ professional development. It was observed, both through responses to tests and through observations of activities, that professionals had greater difficulty in searching for evidence. Participants’level of general knowledge before in-person training was 77.31%, and increased to 80.56% after training.

**Final Considerations:**

with the course, participants’ level of general knowledge increased from regular to good.

## RESUMEN

**Objetivos:**

describir las actividades y repercusiones del curso de capacitación “Objetivos de Desarrollo Sostenible con enfoque en Vigilancia de Enfermedades y Afecciones No Transmisibles en la Amazonía Legal”.

**Métodos:**

estudio observacional y descriptivo. Participaron 36 profesionales de la salud de 14 municipios del estado de Amapá, AP, Brasil. Se aplicaron pruebas previas, posteriores y observaciones (no participante).

**Resultados:**

durante el curso, se desarrollaron actividades de acogida y prácticas educativas y teóricas orientadas a la superación profesional de los participantes. Se observó, tanto a través de las respuestas a las pruebas como de las observaciones de las actividades, que los profesionales tuvieron mayor dificultad en la búsqueda de evidencias. El nivel de conocimientos generales de los participantes del curso antes de la formación presencial era del 77,31%, y aumentó al 80,56% después de la formación.

**Consideraciones Finales:**

con el curso, el nivel de conocimientos generales de los participantes del curso pasó de regular a bueno.

## INTRODUCTION

As a way of renewing and expanding commitments to global sustainability and creating an agenda to replace the Millennium Development Goals (MDGs), in June 2012, in Rio de Janeiro, the United Nations Conference on Sustainable Development, known as Rio+20, took place, resulting in the document “The Future We Want”, which established the foundations for the 193 UN member countries to build a new set of goals and targets for sustainable development, designed to be in force in post-2015 period(1).

Unlike the MDGs, the definition of the Sustainable Development Goals (SDGs), based on the successful experience of the MDGs, was conducted through a broad and democratic process, involving several institutions, civil society organizations and experts(2). On September 25, 2015, the document entitled “Transforming Our World: The 2030 Agenda for Sustainable Development”was adopted, a comprehensive plan of action to promote the well-being of people, the preservation of the planet and the advancement of global prosperity, which includes the 2030 Agenda, a set of 17 SDGs and 169 goals, in effect from January 1, 2016(1).

In summary, the 17 goals were: 1^st –^ no poverty; 2^nd –^ zero hunger; 3^rd –^ good health and well-being; 4^th –^ quality education; 5^th –^ gender equality; 6^th –^ clean water and sanitation; 7^th –^ affordable and clean energy; 8^th^ – decent work and economic growth; 9^th^ – industry, innovation and infrastructure; 10^th –^ reduced inequalities; 11^th –^ sustainable cities and communities; 12^th –^ responsible consumption and production; 13^th –^ climate action; 14^th –^ life below water; 15^th –^ life on land; 16^th –^ peace, justice and strong institutions; and 17^th^ – partnerships for the goals(3).

In Brazil, in 2016, the National Commission for Sustainable Development Goals was created with the purpose of internalizing, disseminating and providing transparency to the process of implementing the UN 2030 Agenda for Sustainable Development in Brazil, with the creation of institutional mechanisms for implementing SDGs, strategies, goals and indicators for monitoring. However, the commission was closed in 2019, with the federal decree that extinguished and limited collegiate bodies of the federal public administration, and was instituted again in 2023(4).

Despite several efforts, with emphasis on the technical studies carried out by both the Brazilian Institute of Geography and Statistics and the Institute of Applied Economic Research, there are factors that impact the fulfillment and national internalization of agreed objectives, such as agenda demobilization at the federal level, starting in 2019, the global political context, the successive crises, among others, which hinder the 2030 Agenda implementation. It is essential to accelerate the resumption of policies that engage public agents, civil society and academia so that the remaining time for implementing the 2030 Agenda is more effective and achieves the agreed objectives and goals(4).

In a study conducted with experts with knowledge of SDGs, they perceived Brazil’s low potential to achieve any of the 17 SDGs, especially 1, 10 and 16, and considered that Brazil should prioritize SDGs 4 and 1 (education and poverty), which they considered the most important and which would help in achieving SDG 3 (health and well-being). These experts also point out that strengthening primary care is crucial(5).

To achieve these global goals, it is also necessary to consider inequalities both between countries and within them. A study that sought to develop a prioritization index to accelerate the achievement of the Brazilian health goals proposed by the 2030 Agenda, assessing the population’s health conditions, showed that northern Brazil has the most vulnerable territories, requiring greater investments in health. In addition to having lower federal resource transfers compared to the national average, the region also has limited management capacity in the municipalities(6).

Considering that Noncommunicable Diseases (NCDs) are one of the greatest global health challenges, causing numerous premature deaths, disabilities and negative impacts on quality of life, in addition to adverse economic effects for families, communities and society, these diseases share socioeconomic determinants and modifiable risk factors, with the possibility for population interventions and public policies focused on their prevention and control as well as implementation of measures that promote social inclusion and reduce inequalities(7). The Strategic Action Plan to Combat Chronic Diseases and Non-Communicable Injuries in Brazil, 2021-2030 (In Portuguese, *Plano de Ações Estratégicas para o Enfrentamento das Doenças Crônicas e Agravos Não Transmissíveis no Brasil, 2021-2030 –* DNCI Plan), in line with SDGs, is presented as a guideline for the prevention of risk factors for Diseases and Non-Communicable Injuries (DNCI) and for promoting the population’ health, establishing 23 indicators and respective targets(8).

Therefore, this study is an excerpt from the project “Strengthening and internalization of the 2030 Agenda of Sustainable Development Goals with the Surveillance of non-communicable diseases and conditions of the State Health Departments of the Legal Amazon region”, funded by the Ministry of Health, which proposed to raise awareness, organize, strengthen and develop internalization strategies in the states of the Legal Amazon region. The project was developed in the nine states of the Legal Amazon (Acre, Amazonas, Amapá, Pará, Maranhão, Mato Grosso, Rondônia, Roraima and Tocantins), under the responsibility of the *Universidade Federal do Tocantins* and the *Universidade Federal do Acre*(9). One of the strategies used was the offering of a training course.

## OBJECTIVES

To describe the activities and repercussions of training course “Sustainable Development Goals with a focus on Surveillance of Non-Communicable Diseases and Injuries in the Legal Amazon”.

## METHODS

### Ethical aspects

The study met all the requirements proposed by Resolution 466/2012 of the Brazilian National Health Council. It was approved by the *Universidade Federal do Tocantins* Research Ethics Committee. The Informed Consent Form was obtained from all individuals involved in the study in writing.

### Study design and setting

This is an observational and descriptive study. The work followed the STrengthening the Reporting of OBservational studies in Epidemiology checklist. Training course“Sustainable Development Goals with a focus on Surveillance of Noncommunicable Diseases and Injuries in the Legal Amazon” was presented at a meeting of the Council of Municipal Health Departments of Amapá. The course aimed to train and/or empower multipliers to internalize SDG agenda in municipalities, with the elaboration and development of intervention projects in health, aiming at the implementation of public policies that are elaborated by healthcare professionals and possibilities of partnerships with other sectors. In the state, the course was coordinated by *Universidade Federal do Amapá* professors.

Municipal health managers were asked to indicate two to three professionals who worked in Health Surveillance or Primary Healthcare management. Prerequisites were to be professionals with higher education who worked in the Health Surveillance Municipal Management and carried out activities directly or indirectly related to the issue of tackling DNCI, in addition to being professionals with higher education who worked in Primary Care management, preferably in activities related to tackling DNCI.

Thirty-six healthcare professionals from 14 municipalities in the state participated (out of a total of 16). The course’s in-person portion had a workload of 16 hours, and took place over two days in August and September 2023. The Student’s Notebook was used to assist in approaching the topics and active learning methodologies. It is worth noting that this notebook was collectively constructed by the general coordination, coordinators and tutors of all the states participating in the general project.

The in-person session covered four thematic learning units: unit I – origin, context, characteristics of SDGs, DNCI Plan and 2030 Agenda convergences with DNCI Plan actions; unit II – translation of knowledge and Evidence-Informed Policies, and search for scientific evidence for health policies; unit III – concept, construction and application of health indicators; unit IV – planning for implementation of health actions, using Situational Strategic Planning (SSP) and advocacy strategy.

### Data collection

A knowledge test was administered upon the arrival of students before starting the training course and at the end of the in-person session. No bibliographical or other student consultation was permitted. There was no time limit for responses. Before the test was given, a brief explanation was given to students, mainly about the course objectives, emphasizing the need for collaboration and voluntary participation.

The test contained 12 questions related to the learning units mentioned above. Of these 12 questions, eight were with affirmative sentences for which students had to mark with an“X”one of the possible alternatives, true (T) or false (F), in which the true statements answered with T or the false ones answered with (F) were considered correct questions and four questions with four alternatives (a, b, c, d), in which they had to mark with an “X” the one considered as the only correct or incorrect question. It is worth noting that the test was constructed based on previously validated theoretical bases: 1) Borges-Andrade’s Integrated and Summative Assessment Model(10); and 2) Abbad’s IMPACT(11). An observation script was used to systematize all the activities carried out in in-person training in detail.

### Data analysis

The data were arranged in Microsoft Office Excel^®^ version 2021 and analyzed using descriptive statistics, being presented in absolute and relative frequencies in a table. Furthermore, based on similar studies, participants’ level of knowledge was classified according to the following scoring diagram: ≤59%,“poor knowledge”; from 60 to 69, “weak”; from 70 to 79, “regular”; from 80 to 89, “good”; from 90 to 99, “very good”; and 100%, classified as “excellent knowledge”(12).

The activity systematization script will be presented descriptively in a chart, containing the learning unit worked on, activity title, the description of what was requested and carried out in the activity, and observations regarding the execution.

## RESULTS

Concerning participant profile, of the 36 participating professionals, it is worth highlighting that 66.67% (n = 24) are nurses; 69.44% (n = 25) are female; 44.44% (n = 16) are between 34 and 42 years old; 52.77% (n = 19) have worked in the health sector for less than six years; 47.22% (n = 17) have a contract as their employment relationship; 58.33% (n = 21) were linked to primary care coordination; 25.0% (n = 9) had been in their current position for less than a year and 41.67% (n = 15) from one to five years in their current position; and 63.89% (n = 23) had only completed higher education, without specialization.

In relation to the course’s impact on participants’knowledge, presented in Table 1, it is clear that students’ general level of knowledge went from regular (77.31%), before the in-person training (according to pre-test), to good (80.56%), after in-person training (according to post-test).

**Table 1:** Knowledge test before and after training course – in-person moment (N = 36), Macapá, Amapá, Brazil, 2023

Item	Question	Pre-test (%)		Post-test (%)	
		Misses	Hits	Misses	Hits
**1.1**	Public policies aligned with sustainable development must consider the economic, environmental, social and institutional dimensions. Answer: True	2.78	97.22	3.03	96.97
**1.2**	Working on the topics outlined in SDGs is a way for municipalities to take care of current and future generations. In this regard, choosing to work on just one goal is enough to ensure that this care is achieved. Answer: False	25.0	75.0	24.24	75.76
**1.3**	According to what is presented in SDG 3, this is not only related to healthcare, but to several other factors, such as the supply of drinking water and sewage systems and healthy food, which indicates a broader vision of health. Answer: True	13.89	86.11	12.12	87.88
**1.4**	SDG 5 “Achieve gender equality and empower all women and girls” is not about addressing health-related issues, but rather about issues of social justice and equality. Answer: False	38.89	61.11	36.36	63.64
**1.5**	The new DNCI Plan expands actions to promote health and combat NCDs, including violence and accidents. Thus, we can understand that this plan contributes to strengthening SDGs. Answer: True	5.56	94.44	3.03	96.97
**2.1**	Evidence-based policy helps policymakers understand these processes and make informed decisions based on the best available scientific evidence, characterized by transparent and systematic access to evidence. Regarding evidence-based approaches to policymaking, select the correct alternative: they allow policymakers to ask critical questions about the available research evidence, demonstrate that they are using good information to inform their decisions, and ensure that assessments of their initiatives are adequate and that the results obtained are realistic and have been previously agreed upon.	41.67	58.33	21.21	78.79
**2.2**	Evidence allows us to clarify public health problems, the characterization, particularities and relevance of the problem and what measures should be taken to solve it. In view of this, mark the incorrect alternative about problem identification = the reflection of knowledge of simultaneous events related to policies and programs only allows us to assess the results of actions.	41.67	58.33	45.45	54.55
**3.1**	Health indicators are used to diagnose the health situation of a community. Based on this statement, select the correct option = indicators are used to measure the impact of health actions on a given population.	16.67	83.33	27.27	72.73
**3.2**	According to Donnabedian (1980), among the types and functions for indicators are those of structure, process and outcome, which are essential for planning and decision-making. According to this statement, mark the CORRECT alternative. The structure indicator answers the questions of what the healthcare service has; the process indicator answers what the healthcare service does; and the outcome indicator answers whether actions were effective and whether the proposal’s general objective was achieved.	30.56	69.44	15.15	84.85
**4.1**	“The mobilization of sectors and social actors to defend an agenda with the aim of giving visibility and causing effects that can be exemplified as the change of a certain policy or the creation of others that meet the interests claimed by the collective” (MORGADO and GOZETTO, 2019) can be considered a definition of advocacy. Answer: True	27.78	72.22	15.15	84.85
**4.2**	SSP consists of four moments for processing problems: explanatory; normative; strategic; and tactical operational. Answer: True	13.89	86.11	21.21	78.79
**4.3**	Health planning has as its main instruments the Health Plan (state/municipal), the Annual Health Program and the Annual Management Report. DNCI Plan is presented as an instrument to support health planning, the definition of investment priorities and the execution with a view to achieving the proposed goals. Answer: True	13.89	86.11	9.09	90.91
	**Result in percentage**	**22.69%**	**77.31%**	**19.44%**	**80.56%**

In pre-test, two questions were classified as poor knowledge (≤59%): questions related to evidence-based policy (2.1 and 2.2). And two questions were classified as weak knowledge (60 to 69%): one question about SDG on gender equality and its relationship with health (1.4), and the other question about types of health indicators (3.2). In post-test, one question about evidence-based policy (2.2) still remained classified as poor knowledge, and one question about gender equality and its relationship with health (1.4) still remained classified as weak knowledge. It is worth noting that, in post-test, the question with the greatest increase in the percentage of correct answers was question 2.1 on“evidence-based approaches in policy formulation”, which went from 58.33% in pre-test to 78.79% in post-test. Also in post-test, of the total of 12 questions, eight questions had an increase in the percentage of correct answers (1.2, 1.3, 1.4, 1.5, 2.1, 3.2, 4.1, 4.3). Of the 12 questions, none showed excellent knowledge (100%) in either preor post-test.

Concerning the activities carried out with participants, for the development of in-person training, there was the support of a large team for execution formed by local coordination, tutor, focal point, volunteer tutor, local project scholarship holder, general coordinator, pedagogical support from the general coordination, nine volunteer academics, a volunteer master’s student, the head and the three people responsible for the State Health Surveillance Superintendence Non-Communicable Diseases and Injuries Unit technical areas. During training, active methodologies were used. In addition to welcoming, 14 activities were developed, with the direct participation of all participants. These activities had a pre-established time, were timed and carried out in groups (one group per municipality). After the conclusion of each activity, the group was asked to present the production of the group to others. Chart 1 described these activities.

**Chart 1:** Summary of training course activities

Unit	Activity	Activity description	Observations
Welcoming	Clothesline of expectations	So that students could express their initial expectations.	All students participated.
Unit I – SDGs and DNCI Plan	Activity 1 – Actions based on SDGs	On the listed websites, students accessed and chose an action based on SDGs of the course. They were asked to identify the following points: what is the topic of the action? What is the objective of the action? Who are the actors involved? What actions were developed? What SDGs were involved? They were asked to perform an analysis of changes and results obtained in the action.	Involvement and interest of the participants, although it was the first time some had contact with SDGs and DNCI Plan.
	Activity 2 – Mind Map based on the topics and actions of DNCI Plan	Using DNCI Plan as a basis for consultation, they were asked to define a central topic and prepare a Mind Map with the actions that best fit the proposal to be constructed.	Objective representations that were easy to present.
	Activity 3 – Characterizing the public health problem	Based on the problem defined for the intervention project by municipality, students should answer the following questions: What is the problem and why can it be considered a public health problem? How was the problem described and what are the consequences of this? How big is the problem? What are the causes of the problem?	The activity was carried out successfully.
	Activity 4 – Prioritizing public health problems	Participants should consider the reality of health data in their municipality, using health situation analysis and the problems highlighted by SDG Agenda and DNCI Plan to build a framework for prioritizing public health problems related to DNCI, following defined criteria and then scoring the public health problems, identifying those of greatest relevance in the municipality.	The activity was easy to carry out and to understand both the problem and its classification.
	Activity 5 – Clothesline of experiences	Based on the prioritized problem, they were asked to consult DNCI Plan and identify the most appropriate actions to solve or minimize it, bringing the group’s experiences and composing a minimum set of four actions that would be developed in the intervention project. *Beginning of the preparation of the intervention project, with the purpose of solving the identified health problem and strengthening the 2030 Agenda in the municipality.	Involvement and interest of the members of each municipality in developing the activity.
	Activity 6 – Intervention project workshop	Considering the health problem that was prioritized by the group for the intervention project to be implemented in the municipality, they were asked to fill in some project items, such as project title, number of participants, target audience, project application location, municipality, health problem and proposed actions.	Participation was active, considering the health problem prioritized to carry out the intervention project to be implemented in the municipalities.
Unit II – EvidenceInformed Policies	Activity 7 – How to organize a roadmap for searching for evidence?	They were equipped to use the platforms to search for evidence.	They performed the activity.
	Activity 8 – Searching for evidence for your intervention project	Based on the information and evidence found, they were asked to write the general objective, the specific objectives and the justification for the project.	They had some difficulty in performing the activity in question because they did not have as much knowledge or experience in handling databases.
Unit III – Health Information Systems and Indicators	Activity 9 – Using indicators to analyze the health situation	Using the example of a fictitious case, they were asked to answer: which indicators could you assume were used in the health situation analysis carried out by the epidemiological surveillance team? Which indicators would you recommend for a more comprehensive analysis of the population profile?	They presented indicators that were consistent with the problems.
	Activity 10 - Choosing indicators for the plan	Considering DNCI Plan and SDGs, they were asked to identify the indicators that could be used in the intervention project and classify them into structure, process or outcome indicators.	They presented indicators that were consistent with the problems, but were not able to differentiate/identify them as indicators of structure, process or outcomes, as requested in the activity.
Unit IV – Planning for Implementation of Health Actions	Activity 11 – Reflecting on the problems	Exercise the explanatory moment and part of SSP regulations. Each group worked on their respective project, developing the following strategies: strategic action 1 – analysis of the problem and identification of its main causes and consequences. Preparation of the problem tree with the three components represented (problem, causes and consequences); strategic action 2 – identification of the critical nodes of the problem. Choose only one critical node to be developed according to the example table, adding the action(s) for its solution.	Some municipalities had difficulty choosing a critical node that had governability.
	Activity 12 – Starting an intervention project	Exercise the strategic and tactical operational moments of SSP. The group carried out a feasibility analysis of the project, using the SWOT matrix, identifying the strengths, weaknesses, threats and opportunities for implementing the project.	The municipalities completed the task.
	Activity 13 – Checklist for the intervention project in the municipality	With their project and a checklist in hand, students had to check the status of each item and try to improve it, filling in the following items: responsible parties; schedule; resources; monitoring; and assessment.	In general, the municipalities had difficulty in constructing the justification, in creating goals and indicators, in indicating those responsible for the actions and in creating the schedule in detail.
	Activity 14 – Applying the advocacy strategy to achieve the project objectives	Based on the project, the groups designed a plan for advocacy actions considering stakeholders from public management, civil society, and the private sector.	The first contact of students with the advocacy strategy.

In relation to positive and negative aspects, the following positive aspects of activities stood out: active participation of students in all activities; use of active methodologies; guidance from the executing team as indispensable support for the development of activities in all groups; reflections and discussions generated; identification of main health problems in municipalities; proposal of actions that can be implemented to solve the problems faced. Regarding the negative aspects, in some activities, pre-established time was not enough. Participants had the greatest difficulties in Unit II (Evidence-Informed Policies), as they were not familiar with the platforms for searching for evidence. After training, a report detailing all activities was sent to the project’s general coordinator, with suggestions for increasing in-person training to three days and reordering activities in Unit II in the Student’s Notebook to facilitate better understanding.

## DISCUSSION

Concerning participant profile, the number of nurses who took the course stands out. From this perspective, nursing needs to strengthen its relationship with SDGs, both scientifically and in decision-making, assuming its active voice and its potential to achieve the goals. It is crucial that nursing highlights what it already does, carries out significant actions and discusses the ways in which it can impact SDGs as a profession, increasing awareness and knowledge of the category about the goals(13,14,15).

In relation to the knowledge of SDGs and DNCI Plan by students, it is clear that these professionals have difficulty in understanding health as a component (directly or indirectly) of all SDGs, complex and crucial in the process of future development, and the importance of health promotion to achieve equity, the empowerment of communities and individuals, in addition to the protection of human rights(16).

The importance of placing health and equity at the center of the political agenda is reinforced, and it is necessary to adopt new governance models that give the health sector the capacity and legitimacy to operate across sectors. To this end, it is essential to strengthen healthcare professionals’ capacities so that they can face this new complexity, understand the various interconnections between sectoral policies and health, and collaborate effectively with other government sectors and stakeholders(17).

A major challenge is the absence of DNCI surveillance area within the Municipal and State Secretariats, which are not even incorporated into their respective institutional organizational charts, with a deficiency in human resources to work in the area, which tends to be even more precarious in smaller municipalities(18).

DNCI Plan seeks to establish and strengthen policies and programs that cover several areas, organize services in an integrated manner, promote efficient governance, generate information to support evidence-based decisions, foster social control and promote innovation in management, research and healthcare services. The aim is to optimize the implementation of proposed actions, improving health surveillance management with innovative resources and institutional structures that favor social participation, governance, shared management, cooperation networks and healthcare service organization(8).

Both tests and observations of activities show that professionals have greater difficulty in seeking evidence. The importance of scientific evidence to inform and support decision-making in health is well known, but it requires informational and technological skills that are not always widely known. For the most part, healthcare professionals have numerous difficulties in using and accessing evidence, mainly related to research skills, lack of structure, time, motivation and excess of information, due to the intense scientific productivity for some topics. Research skills face challenges such as the diversity of sources of information and health research. Some of the search systems are complex, and there are also barriers of a technical, operational, linguistic or even financial nature(19). It is important to note that the health manager must be aware of the impact of disseminated evidence on healthcare(20).

Healthcare professionals need to be equipped for this quest from the moment they are trained and also trained in their places of work to exercise their professions with critical, holistic and human thinking, and to act in a context as complex as the current one, being offered the necessary conditions and tools for this(21).

Concerning knowledge and activities on indicators and Health Information Systems (HIS), the use of HIS optimizes management and care processes, favoring the availability of health information in a timely manner, through the creation of indicators that enable health situation analysis (HSA) at local, state and national levels. The organization of healthcare services must be based on bottom-up planning, from the proximal to the most distal level, based on the population’s HSA. This makes it urgent to have information and indicators that allow the identification of health problems in different territories(22). The development of SDG indicators continues to be a significant challenge for the country, both due to their quantity and diversity(23).

Health managers and teams must be able to develop health action and service planning based on situational analysis, allowing for health situation diagnosis, with adequately systematized information to support decision-making by managers in the territory. However, some difficulties in developing robust and reliable HSA stand out, especially the lack of qualification of professionals, excessive quantity and lack of integration of the different HIS, data quality, underreporting of diseases and injuries, and delay in registration(22).

Regarding planning for the implementation of health actions, there are still challenges for the applicability of strategic planning in the Brazilian Health System (In Portuguese, *Sistema Único de Saúde* - SUS), but these can be faced through the “connection between teaching and service, the co-responsibility of participants and the improvement of this management skill in professionals who work in leadership positions”(24). The advocacy strategy in health is a fundamental axis for professionals’ political action in defense of users’ rights. And actions (social, economic, political and legal) guided by advocacy and organized by committed social actors influence the consolidation of practices, especially from public health policies aimed at improving quality of care and full exercise of users’ rights and for collective health(25).

Finally, we highlighted the use of active methodologies in the training course in the continuing education process, with collective, dynamic and creative activities, using meaningful learning, in which students actively participated in the learning-educating-redoing process. They were leading actors, being able to strengthen the planning of actions consistent with the territory’s reality, enabling reflection and transformation of the work process and professional practices, self-management and institutional change(26,27,28).

### Study limitations

As a limitation of this study, there is the use of test to assess preand post-training knowledge, constructed by the general coordination, which was not applied in previous investigations. However, it was replicated in other states participating in the general project, and may be replicated in other realities, tending to minimize this limitation in order to validate the result and its generalizations. Another limitation is this study addressing only in-person training, due to the magnitude of the project, plurality of actions and diversity of information that will be worked on and presented in future publications.

### Contributions to health and public policies

The results show that the development of SDG objectives and goals in DNCI must be aligned with SUS demands at all levels, in order to create possibilities for their achievement and strengthening in intersectorality, universalization and equity in health, whether in management or, mainly, in assistance, indispensable requirements to meet the diverse and complex scope of 2030 Agenda topics, given the economic, political and cultural, mainly social and environmental determinants of health.

## FINAL CONSIDERATIONS

With the provision of the training course, the general knowledge level of the participants went from regular to good. The strengthening and organization of DNCI surveillance within the scope of the Municipal Health Departments can be encouraged, with continued structuring actions and qualification of epidemiological information, with incentives for state-wide investigations. It is clear that it is important for information to be reliably and easily accessible to managers and healthcare professionals, based on scientific evidence, allowing its timely use in (re)directing actions to tackle DNCI. Knowledge and monitoring of SDGs must be increasingly accessible, not only to the scientific community, but also to professionals, health managers and other sectors, and to society in general. Finally, bringing universities closer to municipal health management and professionals can consolidate health surveillance and promotion actions, in a manner consistent with regional, national and global goals.

## Data Availability

The research data are available within the article.
