# Supplementary material for: Activities and repercussions of a course on Sustainable Development Goals with a focus on surveillance
Source: Rev Bras Enferm. 2025 Oct 27;78(Suppl 1):e20240405. doi: 10.1590/0034-7167-2024-0405 (PMC12563828; doi:10.1590/0034-7167-2024-0405)
Supplement: Translation

# Translation: Atividades e repercussões de um curso sobre Objetivos de Desenvolvimento Sustentável com foco na vigilância

**DOI:** 10.1590/0034-7167-2024-0405pt

**Authors:** Camila Rodrigues Barbosa Nemer, Lethicia Barreto Brandão, Matheus Lopes dos Santos, Gabriella Ferreira Gomes, Anneli Mercedes Celis de Cárdenas, Marta Azevedo dos Santos

**Affiliations:** I Universidade Federal do Amapá. Macapá, Amapá, Brasil; II Universidade Federal do Tocantins. Palmas, Tocantins, Brasil

**Keywords:** Desenvolvimento Sustentável, Doenças não Transmissíveis, Vigilância em Saúde Pública, Atenção Primária à Saúde, Promoção da Saúde

## Abstract

**Objetivos:**

descrever as atividades e repercussões do curso de formação “Objetivos de Desenvolvimento Sustentável com foco na Vigilância de Doenças e Agravos não Transmissíveis na Amazônia Legal”.

**Métodos:**

estudo observacional e descritivo. Participaram 36 profissionais de saúde de 14 municípios do estado do Amapá, AP, Brasil. Foram aplicados pré e pós-testes e observações (não participante).

**Resultados:**

foram desenvolvidas, durante o curso, atividades de acolhimento e práticas educativas e teóricas voltadas para o aprimoramento profissional dos participantes. Observou-se, tanto pelas respostas aos testes quanto pelas observações das atividades, uma maior dificuldade dos profissionais na busca por evidências. O nível de conhecimento geral dos cursistas antes da formação presencial foi de 77,31%, e passou para 80,56% após a formação.

**Considerações Finais:**

com o curso, o nível de conhecimento geral dos cursistas passou de regular para bom.

## INTRODUÇÃO

Como forma de renovar e ampliar os compromissos pela sustentabilidade global e criação de uma agenda de substituição dos Objetivos de Desenvolvimento do Milênio (ODMs), em junho de 2012, no Rio de Janeiro, ocorreu a Conferência das Nações Unidas sobre Desenvolvimento Sustentável, conhecida como Rio+20, resultando no documento“O Futuro que Queremos”, que estabeleceu os alicerces para os 193 países-membros da ONU construírem um novo conjunto de objetivos e metas para o desenvolvimento sustentável, projetados para vigorar no período pós-2015(1).

Diferentemente dos ODMs, a definição dos Objetivos de Desenvolvimento Sustentável (ODS), a partir da experiência exitosa dos ODM, foi conduzida por meio de um processo amplo e democrático, envolvendo diversas instituições, organizações da sociedade civil e especialistas(2). Em 25 de setembro de 2015, foi adotado o documento intitulado “Transformando Nosso Mundo: A Agenda 2030 para o Desenvolvimento Sustentável”, plano abrangente de ação para promover o bem-estar das pessoas, a preservação do planeta e o avanço da prosperidade global, no qual integram a Agenda 2030, um conjunto de 17 ODS e 169 metas, vigentes a partir de 1 de janeiro de 2016(1).

Em resumo, os 17 objetivos foram: 1º – erradicar a pobreza; 2º – combater a fome e promover agricultura sustentável; 3º – garantir saúde e bem-estar; 4º – assegurar educação de qualidade; 5º – alcançar a igualdade de gênero; 6º – garantir água e saneamento para todos; 7º – assegurar energia limpa e acessível; 8º – promover o crescimento econômico sustentável e trabalho decente para todos; 9º – promover infraestruturas resilientes, a industrialização inclusiva e sustentável, e fomentar a inovação; 10º – reduzir a desigualdade dentro dos países e entre eles; 11º – tornar sustentáveis as cidades e comunidades; 12º – assegurar padrões de produção e de consumo responsáveis; 13º – combater a mudança climática e seus impactos; 14º – conservar a vida na água; 15º – proteger, recuperar e promover a vida terrestre; 16º – promover paz, justiça e construir instituições eficazes, responsáveis e inclusivas; e 17º – fortalecer os meios de implementação e a parceria global para o desenvolvimento sustentável(3).

No Brasil, em 2016, foi criada a Comissão Nacional para os Objetivos de Desenvolvimento Sustentável com a finalidade de internalizar, difundir e dar transparência ao processo de implementação da Agenda 2030 para o desenvolvimento sustentável da ONU no Brasil, com a criação de mecanismos institucionais para implementação dos ODS, estratégias, metas e indicadores para acompanhamento. Entretanto, a comissão foi encerrada em 2019, com o decreto federal que extinguiu e limitou órgãos colegiados da administração pública federal, sendo instituída novamente em 2023(4).

Mesmo com vários esforços, com destaque para os estudos técnicos realizados tanto pelo Instituto Brasileiro de Geografia e Estatística quanto pelo Instituto de Pesquisa Econômica Aplicada, há fatores que impactam o cumprimento e a internalização nacional dos objetivos pactuados, como a desmobilização da agenda na esfera federal, a partir de 2019, o contexto político mundial, as sucessivas crises, entre outros, que dificultam a implementação da Agenda 2030. É imprescindível acelerar a retomada das políticas que engajem agentes públicos, sociedade civil e academia, para que o restante do tempo para implementação da Agenda 2030 seja mais eficaz e alcance os objetivos e metas acordados(4).

Em estudo realizado com especialistas com conhecimento sobre os ODS, estes apresentaram percepção de baixo potencial do Brasil para atingir qualquer um dos 17 ODS, especialmente 1, 10 e 16, e consideraram que o Brasil deveria priorizar os ODS 4 e 1 (educação e pobreza), considerados por eles os mais importantes e que ajudariam na consecução do ODS 3 (saúde e bem-estar). Esses especialistas apontam ainda, como crucial, o fortalecimento da atenção básica(5).

Para o alcance dessas metas globais, há que se considerar também as desigualdades tanto entre os países quanto dentro de cada um deles. Estudo que buscou desenvolver um índice de priorização para aceleração do cumprimento das metas brasileiras de saúde propostas pela Agenda 2030, avaliando as condições de saúde da população, evidenciou que a região Norte do Brasil apresenta os territórios mais vulneráveis, necessitando de maiores investimentos em saúde. A região, além de apresentar menor repasses de recursos federais em relação à média nacional, tem também restrita capacidade de gestão nos municípios(6).

Considerando que as Doenças Crônicas Não Transmissíveis (DCNT) são um dos maiores desafios de saúde global, causando numerosos óbitos prematuros, incapacidades e impactos negativos na qualidade de vida, além de efeitos econômicos adversos para famílias, comunidades e a sociedade, essas doenças compartilham determinantes socioeconômicos e fatores de risco modificáveis, com a possibilidade para intervenções populacionais e políticas públicas focadas em sua prevenção e controle, bem como a implementação de medidas que promovam a inclusão social e reduzam as desigualdades(7). O Plano de Ações Estratégicas para o Enfrentamento das Doenças Crônicas e Agravos NãoTransmissíveis no Brasil, 2021-2030 (Plano de Dant), em consonância com os ODS, apresenta-se como diretriz para a prevenção dos fatores de risco das Doenças Crônicas e Agravos Não Transmissíveis (DANT) e para a promoção da saúde da população, estabelecendo 23 indicadores e respectivas metas(8).

Portanto, este estudo é um recorte do projeto“Fortalecimento e interiorização da Agenda 2030 dos Objetivos de Desenvolvimento Sustentável junto à Vigilância de doenças e Agravos não transmissíveis das Secretarias Estaduais de Saúde da região da Amazônia Legal”, financiado pelo Ministério da Saúde, que propunha sensibilizar, organizar, fortalecer e desenvolver estratégias de interiorização nos estados da região da Amazônia Legal. O projeto foi desenvolvido nos nove estados da Amazônia Legal (Acre, Amazonas, Amapá, Pará, Maranhão, Mato Grosso, Rondônia, Roraima e Tocantins), sob responsabilidade da Universidade Federal do Tocantins e da Universidade Federal do Acre(9). Uma das estratégias usadas foi a oferta de um curso de formação.

## OBJETIVOS

Descrever as atividades e repercussões do curso de formação “Objetivos de Desenvolvimento Sustentável com foco na Vigilância de Doenças e Agravos não Transmissíveis na Amazônia Legal”.

## MÉTODOS

### Aspectos éticos

O estudo atendeu todos os requisitos propostos pela Resolução nº 466/2012 do Conselho Nacional de Saúde. Foi aprovado pelo Comitê de Ética em Pesquisa da Universidade Federal do Tocantins. O Termo de Consentimento Livre e Esclarecido foi obtido de todos os indivíduos envolvidos no estudo por meio escrito.

### Desenho e cenário do estudo

Trata-se de estudo observacional e descritivo. O trabalho seguiu o *checklist STrengthening the Reporting of OBservational studies in Epidemiology*. O curso de formação“Objetivos de Desenvolvimento Sustentável com foco na Vigilância de Doenças e Agravos não Transmissíveis na Amazônia Legal” foi apresentado em reunião do Conselho de Secretarias Municipais de Saúde do Amapá. O objetivo do curso era formar e/ou potencializar multiplicadores para interiorização da pauta dos ODS nos municípios, com a elaboração e desenvolvimento de projetos de intervenção da área da saúde, visando à implementação de políticas públicas que sejam elaboradas pelos profissionais de saúde e possibilidades de parceria com outros setores. No estado, o curso estava sob coordenação de docentes da Universidade Federal do Amapá.

Foi solicitada para os gestores de saúde municipais a indicação de dois a três profissionais que atuassem na gestão da Vigilância em Saúde ou Atenção Primária à Saúde. Os pré-requisitos foram ser profissionais de nível superior que atuassem na Gestão Municipal da Vigilância em Saúde e exercer atividades relacionadas direta ou indiretamente à questão do enfrentamento das DANT, além de ser profissionais de nível superior que atuassem na gestão da Atenção Básica, preferencialmente em atividades relacionadas ao enfrentamento das DANT.

Participaram 36 profissionais de saúde de 14 municípios do estado (de um total de 16). O momento presencial do curso teve carga horária de 16 horas, e ocorreu em dois dias nos meses de agosto e setembro de 2023. Foram utilizados Caderno do Cursista para auxiliar a abordagem das temáticas e metodologias ativas de aprendizagem. Destaca-se que este caderno foi construído coletivamente pela coordenação geral, coordenadoras e tutoras de todos os estados participantes do projeto geral.

O momento presencial abordou quatro unidades temáticas de aprendizagem: unidade I – origem, contexto, características dos ODS, Plano de Dant e as convergências da Agenda 2030 com as ações do Plano de Dant; unidade II – tradução do conhecimento e das Políticas Informadas por Evidências, e busca de evidências científicas para políticas de saúde; unidade III – conceito, construção e aplicação de indicadores de saúde; unidade IV – planejamento para implementação de ações em saúde, com uso do Planejamento Estratégico Situacional (PES) e estratégia *advocacy*.

### Coleta de dados

Foi aplicado um teste de conhecimento no momento de chegada dos cursistas antes de iniciar o curso de formação e na finalização do momento presencial, não sendo permitida consulta bibliográfica ou a outro cursista. Não houve limitação de tempo para resposta. Antes da entrega do teste, foi realizada uma rápida explanação aos cursistas, principalmente sobre os objetivos do curso, sendo ressaltada a necessidade de colaboração e participação voluntária.

O teste continha 12 questões relacionadas às unidades de aprendizagem mencionadas anteriormente. Dessas 12 questões, oito eram com frases afirmativas para as quais os cursistas deveriam marcar com um “X” uma das alternativas possíveis, verdadeiro (V) ou falso (F), nas quais as afirmativas verdadeiras respondidas com V ou as falsas respondidas com (F) eram consideradas questões corretas e quatro questões com quatro alternativas (a, b, c, d), nas quais deveriam marcar com um“X”aquela considerada como a única questão correta ou incorreta. Destaca-se que o teste foi construído a partir de bases teóricas já validadas:1) Modelo de Avaliação Integrado e Somativo de Borges-Andrade(10); e 2) IMPACT de Abbad(11). Foi utilizado um roteiro de observação para a sistematização de todas as atividades realizadas na formação presencial de forma detalhada.

### Análise dos dados

Os dados foram dispostos no *software Microsoft Office Excel^®^*, versão 2021, e analisados por meio de estatística descritiva, sendo apresentados em frequências absolutas e relativas em tabela. Ademais, embasado em estudo semelhantes, o nível de conhecimento dos participantes foi classificado segundo o seguinte diagrama de pontuação: ≤59%,“conhecimento pobre”; de 60 a 69, “fraco”; de 70 a 79,“regular”; de 80 a 89,“bom”; de 90 a 99,“muito bom”; e 100%, classificado como “conhecimento excelente”(12).

O roteiro de sistematização das atividades será apresentado de forma descritiva em quadro, contendo a unidade de aprendizagem trabalhada, o título da atividade, a descrição do que foi solicitado e realizado na atividade, e as observações quanto à execução.

## RESULTADOS

Quanto ao perfil dos participantes, dos 36 profissionais participantes, destaca-se que 66,67% (n = 24) são enfermeiros; 69,44% (n = 25) são do sexo feminino; 44,44% (n = 16) estão na faixa etária entre 34 e 42 anos; 52,77% (n = 19) trabalhavam no setor da saúde há menos de seis anos; 47,22% (n = 17) têm como vínculo empregatício o contrato; 58,33% (n = 21) estavam vinculados à coordenação de atenção primária; 25,0% (n = 9) estavam a menos de um ano no cargo atual, e 41,67% (n = 15), de um a cinco anos no cargo atual; e 63,89% (n = 23) tinham apenas o superior completo, sem especialização.

Quanto às repercussões do curso no conhecimento dos participantes, apresentadas na Tabela 1, evidencia-se que o nível de conhecimento geral dos cursistas passou de regular (77,31%), antes da formação presencial (segundo o pré-teste), para bom (80,56%), após a formação presencial (segundo o pós-teste).

**Tabela 1:** Teste de conhecimento pré e pós-curso de formação – momento presencial (N = 36), Macapá, Amapá, Brasil, 2023

Item Questão	Pré-teste (%)		Pós-teste (%)	
	Erros	Acertos	Erros	Acertos
**1.1**	Políticas públicas alinhadas ao desenvolvimento sustentável devem considerar as dimensões econômica, ambiental, social e institucional. Resposta: Verdadeira	2,78	97,22	3,03	96,97
**1.2**	Trabalhar os temas previstos nos ODS é um caminho para que os municípios possam cuidar da geração atual e futura. Neste sentido, a escolha de trabalhar apenas um objetivo já é suficiente para garantir o alcance deste cuidado. Resposta: Falsa	25,0	75,0	24,24	75,76
**1.3**	De acordo com o que é apresentado no ODS 3, este não está relacionado apenas à assistência à saúde, mas a diversos outros fatores, como a oferta de água potável e de sistemas de esgotamento sanitário e alimentação saudável, o que indica uma visão ampliada da saúde. Resposta: Verdadeira	13,89	86,11	12,12	87,88
**1.4**	O ODS 5 “Alcançar a igualdade de gênero e empoderar todas as mulheres e meninas” não está relacionado ao enfrentamento de problemas relativos à saúde, e sim a questões de justiça e igualdade social. Resposta: Falsa	38,89	61,11	36,36	63,64
**1.5**	O novo Plano de Dant amplia as ações de promoção à saúde e de enfrentamento das DCNT, incluindo violências e acidentes. Assim, podemos entender que este plano contribui para o fortalecimento dos ODS. Resposta: Verdadeira	5,56	94,44	3,03	96,97
**2.1**	A política baseada em evidências científicas ajuda os formuladores de política a entender esses processos e realizar tomada de decisão fundamentada pela melhor evidência científica disponível, caracterizada pelo acesso transparente e sistemático às evidências. Quanto às abordagens embasadas em evidências na formulação de políticas, marque a alternativa correta: permitem que os formuladores de políticas façam perguntas críticas sobre a evidência da pesquisa disponível, demonstrem que estão usando boas informações para fundamentar suas decisões, e assegurem que as avaliações das suas iniciativas são adequadas e que os resultados obtidos são realistas e foram previamente acordados.	41,67	58,33	21,21	78,79
**2.2**	As evidências permitem esclarecer problemas de saúde pública, a caracterização, particularidades e relevância do problema e quais medidas devem ser tomadas para resolvê-lo. Diante disso, marque a alternativa incorreta sobre a identificação de problemas: a reflexão de um conhecimento de eventos simultâneos relacionados a políticas e programas permite apenas avaliar o resultado das ações.	41,67	58,33	45,45	54,55
**3.1**	Os indicadores de saúde são utilizados para o diagnóstico da situação de saúde de uma comunidade. Com base nesta afirmação, assinale a opção correta: os indicadores são utilizados para medir o impacto das ações de saúde em uma determinada população.	16,67	83,33	27,27	72,73
**3.2**	Segundo Donnabedian (1980), entre os tipos e funções para indicadores, encontram-se os de estrutura, de processo e de resultado, sendo estes indispensáveis para o planejamento e tomada de decisão. De acordo com esta afirmativa, marque a alternativa CORRETA. O indicador de estrutura responde às questões do que o serviço de saúde tem; o indicador de processo responde sobre o que o serviço de saúde faz; e o indicador de resultado responde se as ações foram efetivas e se o objetivo geral da proposta foi alcançado.	30,56	69,44	15,15	84,85
**4.1**	“A mobilização de setores e atores sociais para a defesa de uma pauta com o objetivo de dar visibilidade e provocar efeitos que podem ser exemplificados como a mudança de determinada política ou a criação de outras que atendam aos interesses reivindicados pelo coletivo” (MORGADO e GOZETTO, 2019) pode ser considerado uma definição de *advocacy*. Resposta: Verdadeira	27,78	72,22	15,15	84,85
**4.2**	O PES é composto por quatro momentos para o processamento dos problemas: explicativo; normativo; estratégico; e tático operacional. Resposta: Verdadeira	13,89	86,11	21,21	78,79
**4.3**	O planejamento em saúde tem como instrumentos principais o Plano de Saúde (estadual/municipal), a Programação Anual de Saúde e o Relatório Anual de Gestão. O Plano de Dant apresenta-se como instrumento de apoio ao planejamento em saúde, à definição de prioridades para investimento e à execução com vistas ao alcance das metas propostas. Resposta: Verdadeira	13,89	86,11	9,09	90,91
	**Resultado em porcentagem**	**22,69%**	**77,31%**	**19,44%**	**80,56%**

No pré-teste, duas questões foram classificadas como conhecimento pobre (≤59%): as questões relacionadas à política baseada em evidências (2.1 e 2.2). E duas questões foram classificadas como conhecimento fraco (de 60 a 69%): uma questão sobre ODS de igualdade de gênero e a relação com a saúde (1.4), e a outra questão sobre tipos de indicadores de saúde (3.2). No pós-teste, uma questão sobre política baseada em evidências (2.2) ainda permaneceu classificada como conhecimento pobre, e uma questão sobre igualdade de gênero e a relação com a saúde (1.4) permaneceu ainda classificada como conhecimento fraco. Destaca-se que, no pós-teste, a questão com maior aumento da porcentagem de acertos foi a questão 2.1 sobre “abordagens embasadas em evidências na formulação de políticas”, que passou de 58,33%, no pré-teste, para 78,79%, no pós-teste. Ainda no pós-teste, do total das 12 questões, oito questões tiveram o aumento da porcentagem de acertos (1.2, 1.3, 1.4, 1.5, 2.1, 3.2, 4.1 , 4.3). Das 12 questões, nenhuma apresentou conhecimento excelente (100%) tanto no pré quanto no pós-teste.

Quanto às atividades realizadas com os participantes, para o desenvolvimento da formação presencial, contou-se com o apoio de uma grande equipe para execução formada por coordenação local, tutora, ponto focal, tutora voluntária, bolsista local do projeto, coordenadora geral, apoio pedagógico da coordenação geral, nove acadêmicos voluntários, mestrando voluntário, a chefe e as três responsáveis das áreas técnicas da Unidade de Doenças e Agravos Não Transmissíveis da Superintendência de Vigilância em Saúde do estado. Durante a formação, foram utilizadas metodologias ativas. Foram desenvolvidas, além do acolhimento, 14 atividades, com a participação direta de todos os cursistas. Essas atividades tinham tempo pré-estabelecido, sendo cronometradas e realizadas em grupos (um grupo por município). Após a conclusão de cada atividade, era solicitada a apresentação da produção do grupo aos demais. Tais atividades estão descritas no Quadro 1.

**Quadro 1:** Síntese das atividades do curso de formação

Unidade	Atividade	Descrição da atividade	Observações
Acolhimento	Varal de expectativas	Para que os cursistas pudessem expressar suas expectativas iniciais.	Todos os cursistas participaram.
Unidade I – ODS e Plano de Dant	Atividade 1 – Ações baseadas nos ODS	Em *sites* listados, os cursistas acessaram e escolheram uma ação baseada nos ODS do curso. Foi solicitado que identificassem os seguintes pontos: qual o tema da ação? Qual o objetivo da ação? Quem são os atores envolvidos? Quais as ações desenvolvidas? Quais os ODS envolvidos? Foi solicitado que realizassem uma análise das mudanças e resultados obtidos na ação.	Envolvimento e interesse dos cursistas, embora fosse o primeiro contato de alguns sobre ODS e Plano de Dant.
	Atividade 2 – Mapa Mental baseado nos tópicos e ações do Plano de Dant	Tendo como base de consulta o Plano de Dant, foi solicitado que definissem um tópico central e elaborassem um Mapa Mental com as ações que mais se encaixam na proposta a ser construída.	Representações objetivas com facilidade de exposição.
	Atividade 3 – Caracterizando o problema de saúde pública	Com base no problema definido para o projeto de intervenção por município, os cursistas deveriam responder às questões abaixo: qual é o problema e por que ele pode ser considerado um problema de saúde pública? Como o problema foi descrito e quais são as consequências disso? Quão grande é o problema? Quais as causas do problema?	Atividade realizada com êxito.
	Atividade 4 - Priorizando problemas de saúde pública	Os cursistas deveriam considerar a realidade dos dados em saúde do seu município, utilizando análise de situação de saúde e os problemas apontados pela Agenda dos ODS e pelo Plano de Dant para construir um quadro de priorização de problemas de saúde pública relacionados às DANT, seguindo critérios definidos e, em seguida, pontuando os problemas de saúde pública, identificando aqueles de maior relevância no município.	Atividade de fácil execução e compreensão tanto do problema quanto da sua classificação.
	Atividade 5 – Varal de experiências	A partir do problema priorizado, foi solicitado que consultassem o Plano de Dant e identificassem as ações mais adequadas para sua solução ou minimização, trazendo as experiências do grupo e compondo um conjunto mínimo de quatro ações que seriam desenvolvidas no projeto de intervenção. *Início da elaboração do projeto de intervenção, com a finalidade de resolver o problema de saúde identificado e fortalecer a Agenda 2030 no município.	Envolvimento e interesse dos integrantes de cada município para o desenvolvimento da atividade.
	Atividade 6 – Oficina de projeto de intervenção	Considerando o problema de saúde que foi priorizado pelo grupo para o projeto de intervenção a ser implementado no município, foi solicitado que preenchessem alguns itens do projeto, como título do projeto, número de participantes, público-alvo, local de aplicação do projeto, município, problema de saúde e ações propostas.	A participação foi ativa, considerando o problema de saúde priorizado para realizar o projeto de intervenção a ser implementado nos municípios.
Unidade II – Políticas Informadas por Evidências	Atividade 7 – Como organizar um roteiro para a busca de evidências?	Foram instrumentalizados para utilização das plataformas para busca de evidências.	Realizaram a atividade.
	Atividade 8 – Buscando evidências para o seu projeto de intervenção	A partir das informações e das evidências encontradas, foi solicitado que escrevessem o objetivo geral, os objetivos específicos e a justificativa do projeto.	Apresentaram certa dificuldade em executar a atividade em questão por não terem tanto conhecimento quanto prática no manuseio das bases de dados.
Unidade III – Indicadores e Sistemas de Informação em Saúde	Atividade 9 – Usando os indicadores para análise de situação de saúde	Usando o exemplo de um caso fictício, foi solicitado que respondessem: quais indicadores poderiam pressupor que foram utilizados na análise de situação de saúde realizada pela equipe de vigilância epidemiológica? Quais indicadores você recomendaria para uma análise mais ampliada do perfil da população?	Apresentaram indicadores condizentes com os problemas.
	Atividade 10 - Escolhendo indicadores para o plano	Considerando o Plano de Dant e os ODS, foi solicitado que identificassem os indicadores que poderiam ser utilizados no projeto de intervenção e classificassem em indicadores de estrutura, de processo ou de resultados.	Apresentaram indicadores condizentes com os problemas, contudo não foram capazes de diferenciar/identificar como indicadores de estrutura, de processo ou de resultados, conforme solicitado na atividade.
Unidade IV – Planejamento para Implementação de Ações em Saúde	Atividade 11 – Refletindo sobre os problemas	Exercitar o momento explicativo e parte do normativo do PES. Cada grupo trabalhou seu respectivo projeto, sendo desenvolvidas as seguintes estratégias: ação estratégica 1 – análise do problema e identificação de suas principais causas e consequências. Elaboração da árvore de problemas com os três componentes representados (problema, causas e consequências); ação estratégica 2 – identificação dos nós críticos do problema. Escolha apenas um nó crítico para ser desenvolvido conforme quadro exemplo, acrescido da(s) ação(ões) para sua solução.	Alguns municípios apresentaram dificuldades de escolher um nó crítico que tivesse governabilidade.
	Atividade 12 – Iniciando um projeto de intervenção	Exercitar os momentos estratégico e tático operacional do PES. O grupo realizou a análise de viabilidade do projeto, usando a matriz *SWOT*, com identificação das forças, fraquezas, ameaças e oportunidades para implementação do projeto.	Os municípios cumpriram a tarefa.
	Atividade 13 – *Checklist* do projeto de intervenção no município	De posse do seu projeto e de um *checklist*, os cursistas tinham que verificar a situação de cada item e tentar melhorá-lo, preenchendo os seguintes itens: responsáveis; cronograma; recursos; monitoramento; e avaliação.	De forma geral, os municípios apresentaram dificuldades na construção da justificativa, na construção de metas e indicadores, em indicar os responsáveis pelas ações e construir o cronograma detalhadamente.
	Atividade 14 - Aplicando a estratégia *advocacy* para o alcance dos objetivos do projeto	A partir do projeto, os grupos desenharam um planejamento para ações de *advocacy* considerando os atores da gestão pública, da sociedade civil e da iniciativa privada.	O primeiro contato dos cursistas com a estratégia *advocacy*.

Quanto aos pontos positivos e negativos, os seguintes pontos positivos da realização das atividades se destacaram: a participação ativa dos cursistas em todas as atividades; o uso de metodologias ativas; a orientação da equipe executora como apoio indispensável para o desenvolvimento das atividades em todos os grupos; as reflexões e discussões geradas; a identificação dos principais problemas de saúde dos municípios; a proposição de ações que possam ser implementadas para a resolutividade dos problemas enfrentados. Sobre os pontos negativos, em algumas atividades, o tempo pré-estabelecido não foi o suficiente. Os cursistas apresentaram maiores dificuldades na Unidade II (Políticas Informadas por Evidências), por não terem familiaridade com as plataformas para busca das evidências. Após a formação, foi enviado à coordenação geral do projeto um relatório com todas as atividades de forma detalhada, com sugestões de aumento da formação presencial para três dias e reordenamento das atividades na Unidade II no Caderno do Cursista para facilitar melhor a compreensão.

## DISCUSSÃO

Quanto ao perfil dos cursistas, destaca-se a quantidade de enfermeiros que fizeram o curso. Nessa perspectiva, a enfermagem precisa fortalecer sua relação com os ODS, tanto cientificamente quanto na tomada de decisão, assumindo sua voz ativa e seu potencial para a consecução dos objetivos. Mostra-se crucial que a enfermagem evidencie o que já realiza, execute ações significativas e discuta as formas de impacto enquanto profissão sobre os ODS, aumentando a conscientização e conhecimento da categoria sobre os objetivos(13,14,15).

Em relação ao conhecimento sobre ODS e Plano de Dant pelos cursistas, percebe-se que esses profissionais têm dificuldade em compreender a saúde como componente (direta ou indiretamente) de todos os ODS, complexa e crucial no processo de desenvolvimento futuro, e a importância da promoção da saúde para alcançar a equidade, o empoderamento das comunidades e dos indivíduos, além da proteção dos direitos humanos(16).

Reforça-se a importância de colocar a saúde e a equidade no foco central da agenda política, sendo necessário adotar novos modelos de governança que ofereçam ao setor saúde a capacidade e legitimidade para operar em todos os setores. Para isso, é essencial fortalecer as capacidades dos profissionais de saúde para que possam enfrentar essa nova complexidade, entender as diversas interligações entre políticas setoriais e saúde, e colaborar de forma eficaz com outros setores governamentais e partes interessadas(17).

Um grande desafio é a ausência da área de vigilância de DANT dentro das Secretarias Municipais e Estaduais, que sequer são incorporadas aos respectivos organogramas institucionais, com deficiência nos recursos humanos para atuarem na área, o que tende a ser ainda mais precário em municípios de menor porte(18).

Com o Plano de Dant, busca-se estabelecer e fortalecer políticas e programas que abranjam várias áreas, organizar serviços de forma integrada, promover uma governança eficiente, gerar informações para embasar decisões com base em evidências, fomentar o controle social e promover inovação na gestão, pesquisa e serviços de saúde. Busca-se otimizar a implementação das ações propostas, melhorando a gestão da vigilância em saúde com recursos inovadores e estruturas institucionais que favoreçam a participação social, a governança, a gestão compartilhada, as redes de cooperação e a organização dos serviços de saúde(8).

Evidencia-se, tanto pelos testes quanto pelas observações das atividades, uma maior dificuldade dos profissionais na busca por evidências. Sabe-se da importância das evidências científicas para informar e subsidiar a tomada de decisão em saúde, exigindo, entretanto, competências informacionais e tecnológicas que nem sempre são amplamente conhecidas. Em grande parte, os profissionais de saúde têm inúmeras dificuldades para utilização e acesso, principalmente relacionadas às habilidades de pesquisa, falta de estrutura, tempo, motivação e excesso de informações, devido à intensa produtividade científica para algumas temáticas. As habilidades de pesquisa enfrentam desafios, como a diversidade de fontes de informação e pesquisa em saúde. Alguns dos sistemas de busca são complexos, e existem também barreiras de natureza técnica, operacional, linguística ou mesmo financeira(19). Ressalta-se que o gestor de saúde deve estar ciente do impacto das evidências disseminadas na assistência em saúde(20).

Os profissionais de saúde precisam ser instrumentalizados para essa busca desde sua formação e capacitados também em seus locais de serviço para exercerem suas profissões com pensamento crítico, holístico e humano, e atuarem em um contexto tão complexo como o atual, sendo ofertadas as condições e ferramentas necessárias para tal(21).

Quanto ao conhecimento e às atividades sobre indicadores e Sistemas de Informação em Saúde (SIS), a utilização dos SIS otimiza os processos gerenciais e assistenciais, favorecendo a disponibilidade de informações de saúde em tempo hábil, através da criação de indicadores que possibilitem a análise de situação de saúde (ASIS) nos níveis local, estadual e nacional. É necessário que a organização dos serviços de saúde seja baseada no planejamento ascendente, desde o nível proximal até o mais distal, fundamentado na ASIS da população. Isso torna urgente a disponibilidade de informações e indicadores que permitam identificar os problemas de saúde nos diferentes territórios(22). A elaboração dos indicadores dos ODS continua sendo um desafio significativo para o país, tanto devido à sua quantidade quanto devido à sua diversidade(23).

Os gestores e equipes de saúde devem estar aptos para desenvolver o planejamento de ações e serviços de saúde com base na análise situacional, permitindo o diagnóstico situacional de saúde, com informações sistematizadas, de forma adequada, para subsidiar a tomada de decisão de gestores no território. Porém, destacam-se algumas dificuldades na elaboração de ASIS robustas e fidedignas, com destaque para a falta de qualificação dos profissionais, quantidade exacerbada e não integração dos diferentes SIS, qualidade dos dados, subnotificação de doenças e agravos, e demora dos registros(22).

Quanto ao planejamento para implementação de ações em saúde, ainda há desafios para aplicabilidade do planejamento estratégico no Sistema Único de Saúde (SUS), porém podendo ser enfrentados por meio da “conexão ensino e serviço, da corresponsabilidade dos participantes e da aprimoração desta habilidade de gestão em profissionais que atuam em posições de liderança”(24). A estratégia *advocacy* na saúde configura-se como um eixo fundamental para ação política dos profissionais em defesa dos direitos dos usuários. E ações (sociais, econômicas, políticas e jurídicas) norteadas pelo *advocacy* e organizadas por atores sociais comprometidos influenciam a consolidação de práticas, especialmente a partir de políticas públicas de saúde voltadas à melhor qualidade da assistência e pleno exercício do direito dos usuários e para a saúde coletiva(25).

Destaca-se, por fim, o uso de metodologias ativas no curso de formação no processo de educação permanente, com atividades coletivas, dinâmicas e criativas, utilizando-se a aprendizagem significativa, no qual os cursistas agiram ativamente no processo de aprender-educar-refazer. Eles foram protagonistas, podendo fortalecer o planejamento de ações coerentes com a realidade do território, possibilitando a reflexão e transformação do processo de trabalho e das práticas profissionais, autogestão e mudança institucional(26,27,28).

### Limitações do estudo

Como limitação do estudo, tem-se a utilização do teste para avaliar o conhecimento pré e pós-formação, construído pela coordenação geral, que não foi aplicado em investigações anteriores. Entretanto, foi replicado nos outros estados participantes do projeto geral, e pode vir a ser replicado em outras realidades, tendendo a minimizar essa limitação de forma a validar o resultado e suas generalizações. Outra limitação é a apresentação neste estudo apenas da formação presencial, devido à grandeza do projeto, pluralidade de ações e diversidade de informações que serão trabalhadas e apresentadas em futuras publicações.

### Contribuições para as áreas da saúde e políticas públicas

Os resultados evidenciam que o desenvolvimento dos objetivos e metas dos ODS nas Dant deve ser alinhado de acordo com as demandas do SUS em todos seus níveis, de modo a criar possibilidades para seu alcance e fortalecimento na intersetorialidade, universalização e equidade em saúde, seja na gestão ou, principalmente, na assistência, requisitos indispensáveis para atender à diversa e complexa abrangência dos temas da Agenda 2030, diante dos determinantes econômicos, políticos e culturais, principalmente sociais e ambientais da saúde.

## CONSIDERAÇÕES FINAIS

Com a oferta do curso de formação, o nível de conhecimento geral dos cursistas passou de regular para bom. Pode-se incentivar o fortalecimento e organização da vigilância das Dant no âmbito das Secretarias Municipais de Saúde, com continuidade de ações estruturantes e qualificação das informações epidemiológicas, com incentivo a investigações de abrangência estadual. Mostra-se evidente a importância de as informações estarem fidedignamente e facilmente acessíveis para gestores e profissionais de saúde, fundamentada em evidências científicas, permitindo seu uso oportuno no (re)direcionamento de ações de enfrentamento das Dant. O conhecimento e monitoramento sobre os ODS precisam ser cada vez mais acessíveis, não só para a comunidade científica, mas também para profissionais, gestores de saúde e de outros setores, e para a sociedade em geral. Por fim, a aproximação da universidade com a gestão e profissionais da assistência dos municípios pode consolidar ações de vigilância e de promoção de saúde, de forma coerente com metas regionais, nacionais e globais.

## Data Availability

Os dados da pesquisa estão disponíveis no corpo do artigo.
